# Routine monitoring systems for integrated community case management programs: Lessons from 18 countries in sub–Saharan Africa

**DOI:** 10.7189/jogh-04-020301

**Published:** 2014-12

**Authors:** Tanya Guenther, Yolanda Barberá Laínez, Nicholas P Oliphant, Martin Dale, Serge Raharison, Laura Miller, Geoffrey Namara, Theresa Diaz

**Affiliations:** 1 Save the Children, Washington DC, USA; 2 International Rescue Committee, New York, NY, USA; 3 UNICEF, Programme Division, Health, New York, NY, USA; 4 Population Services International, Nairobi, Kenya; 5 John Snow Inc., Arlington, VA, USA; 6 International Rescue Committee, Freetown, Sierra Leone; 7 Malaria Consortium, Kampala, Uganda

Integrated community case management (iCCM) programs are expanding rapidly in many low– and middle–income countries, particularly in sub–Saharan Africa. Conclusions from the recent review of iCCM programs in Africa emphasized the critical importance of using routine data to assess program performance and to inform impact evaluations [1]. Yet monitoring systems often fail to deliver quality data (defined as relevant, complete, timely and accurate [2]) and program managers do not have the capacity or are not empowered to use data for decision–making and corrective action [3]. Monitoring systems for iCCM suffer from many of the same shortcomings of the broader routine health information systems (HIS), but extending these systems to the community level at scale presents unique challenges and constraints. While the literature highlighting results of iCCM programs has expanded, little has been published that explores the monitoring systems necessary to support successful implementation.

This paper aims to synthesize lessons learned from recent experience developing and implementing systems for routine monitoring of large scale iCCM programs. These lessons were compiled from the primary partners supporting iCCM implementation across 18 countries in sub–Saharan Africa through interviews with monitoring focal persons and review of relevant documents and tools and informed by literature on strengthening routine health information systems more broadly [3–5]. We first outline the rationale for routine data and the challenges iCCM programs face to establish functional monitoring systems to generate such data. We then characterize the current state of routine monitoring systems for iCCM, summarize lessons learned and conclude with a way forward.

## WHY IS ROUTINE MONITORING DATA SO IMPORTANT FOR iCCM PROGRAMS?

Children fall ill with iCCM conditions multiple times over the course of a year (estimated to range between 3.3 episodes of diarrhea [6], 1.7 episodes of malaria [7] and 0.3 episodes of pneumonia in sub–Saharan Africa [6]). Health services need to be routinely available and accessible to provide timely and appropriate treatment. Currently, the gold standard for measuring treatment coverage is through household surveys such as the Demographic and Health Surveys (DHS) and Multiple Indicator Cluster Surveys (MICS) [8]. However, household surveys are resource intensive and typically capture data on care–seeking and treatment practices for only a two week recall period [8,9]. This timeframe is insufficient to capture performance over actual project cycles or long implementation periods, as coverage is sensitive to fluctuations in supply side factors (availability of providers, medicines and supplies), demand side factors (ability to cover transport or other associated costs, opportunity costs for family to seek care, awareness and perception of services) and contextual factors (seasonality, flooding, conflicts) [10]. Further, the validity of household survey questions to measure appropriate treatment coverage for pneumonia and malaria has been called into question; the small sample sizes for many conditions preclude precise estimates, especially at subnational level; and data on source of treatment are not always collected [9]. In addition, the samples used in most widely available household surveys, such as DHS and MICS, are often representative of the national or regional population, making it difficult for district managers to extract useful information for program monitoring. To better understand the contribution of iCCM and to improve implementation, program implementers, managers and evaluators require real–time, sound data that enables tracking trends over time on factors associated with high coverage, quality and cost–efficiency, such as rates of treatment, supervision and medicine availability [11].

**Figure:**
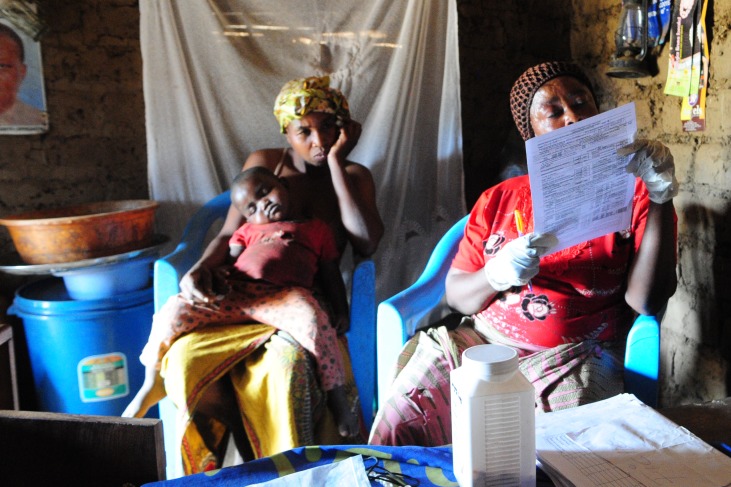
Photo: Courtesy of Yolanda Barberá Laínez, International Rescue Committee

## WHAT CHALLENGES DO WE FACE FOR ROUTINE MONITORING OF iCCM?

Building and maintaining systems to effectively monitor iCCM implementation at scale is inherently complex, involving data collection from thousands of multi–tasked community health workers (CHWs), who in many cases are volunteers with limited formal education. While community health information systems share characteristics and shortcomings with the broader routine HIS of which they are part of (or should be part of), the complexity of increasing the number, diversity and geographic dispersion of service delivery points creates several unique challenges. Consider Rwanda, where an estimated 30 000 CHWs are providing iCCM services and generating data on a monthly basis – more than 50 times the number of public health facilities (576 in 2014 according to the Ministry of Health (MOH); personal communication; Ministry of Health Rwanda). There is often wide variation in levels of literacy and numeracy among CHWs even within a single country. In Uganda, CHWs in central districts are generally literate and numerate, while CHWs in western districts are semi–literate and those in remote northern districts are mostly illiterate. The timeline required for collecting reports from a large network of iCCM CHWs, who are by definition far from health facilities, is also greater in most settings; one cannot expect it will take the same amount of time to receive reports from a large set of CHWs as for health facility reports within a given administrative unit. Monitoring systems need to accommodate this scale and variation, and yet the window of time to develop procedures and tools (registers, reporting and compilation forms, data management processes, training materials) is typically very short with limited opportunity for testing and refinement. The costs of printing tools and retraining the thousands of CHWs and first level health workers each time a register or report is revised are prohibitive.

By design, community case management strategies target underserved areas with limited access to formal health services; not surprisingly, these areas typically also experience poor physical infrastructure (inadequate roads, limited transportation options, minimal electricity, spotty if any internet or mobile network coverage) and an already overstretched health system with inadequate human resources for supervision and monitoring. Moreover, in most countries, multiple donors and agencies are supporting iCCM implementation and have their own short–term reporting requirements that often do not meet country information needs or take into account the underlying system capacities or constraints [3]. This creates a tendency to impose far greater documentation and reporting requirements on CHWs than is expected at the facility level, putting pressure on the lowest level of the system and compromising data quality and completeness. Furthermore, there are limited incentives for partners to invest in strengthening a national system for routine monitoring, which requires time, compromise, priority setting and coordination. As such, in many cases routine monitoring data are often undervalued and marginalized in favor of periodic surveys over which donors and implementing partners can exert greater control.

Finally, whereas the technical content for an iCCM program can be relatively standard across settings and indicator definitions can and should adhere to international standards, there is no “one–size–fits–all” approach for *how* to implement an effective monitoring system for iCCM. Approaches must be tailored for each context and be light and flexible enough to adjust to rapidly changing program contexts.

## WHAT IS THE CURRENT STATUS OF ROUTINE MONITORING SYSTEMS FOR iCCM?

While iCCM programs are progressing to scale rapidly, monitoring systems are lagging behind in strengthening the six functional components required for a HIS to generate quality information as outlined by the Health Metrics Network Framework [12]. **Table 1** contrasts the typical state of monitoring systems for iCCM with the ideal situation and demonstrates that monitoring systems for iCCM still need to improve. These issues are not unique to iCCM [4]. Few countries have strategic plans for their HIS and fewer still have annual, costed plans to operationalize them. Very few MOH have M&E staff explicitly responsible for iCCM data, and where they do exist, they are usually short–term secondments supported by partners. National M&E plans with prioritized indicators for iCCM are lacking in most countries with large scale iCCM programs and those countries with plans have struggled to operationalize them. Reporting systems tend to be burdensome and CHWs are often asked to record and report vast quantities of data that are rarely used and could be obtained more effectively and efficiently from other sources. In many countries, CHWs deliver more than just iCCM, but monitoring and supervision systems are set up vertically. To our knowledge, only a handful of countries (Ghana, Uganda, Zambia, Malawi, Mali, and Niger) have initiated capture of data on community treatments in their national HIS and even in these cases, the data are not fully compatible with facility level data making it difficult to measure the proportion of total treatments provided through iCCM to assess whether the program is expanding coverage as intended. Parallel data collection and reporting systems are commonplace and procedures to convert raw data into user–friendly information products and disseminate to decision–makers are generally absent.

**Table 1:** Characterization of M&E for iCCM according to components of health information systems

HIS system components	Typical situation for ICCM M&E	Ideal situation
Health information system resources	• Lack of M&E and data management staff within MOH with clear roles, responsibilities and accountability for iCCM specified within job descriptions • Inadequate human resource capacity to ensure timely and quality data collection, reporting, management, analysis and use	• Trained staff within MOH with clear roles and responsibilities to manage iCCM monitoring data • Support mechanisms in place to provide ongoing mentoring and refresher training • Costed annual plan for health information systems including iCCM data needs
Indicators	• Weak or non–existent national plan for monitoring and evaluating iCCM • Use of non–standard indicators; proliferation of indicators that differ across donors and implementing partners	• Clear national plan for M&E of iCCM (as a part of a broader strategy and costed annual plan for health information systems) • Prioritization of limited number of indicators that are harmonized across MOH, donors and implementing partners as part of a standardized minimum core set for the HIS
Data sources	• Complicated registers and reporting tools that are burdensome for users and/or too costly for use at national scale (eg, color registers or too many registers) • Lack of standardized tools across partners • Limited integration and coordination with other programs/interventions implemented by CHWs • Fragmented use of information communication technology (ICT) and mHealth solutions	• User–centered, low cost, standardized tools that are appropriate for the literacy and numeracy level of the health workers, capture limited set of data elements linked to priority indicators, and can be produced at scale • MOH–coordinated use of appropriate ICT and mHealth solutions that can be scaled up
Data management	• Suboptimal capacity of information systems (HIS/LMIS) to meet needs for data management, analysis, visualization, sharing and learning • Community treatment data not integrated into national HIS or not disaggregated by point of service • Implementing partners maintaining parallel reporting systems • Lack of mechanisms to periodically assess quality of ICCM data (through audits and triangulation with other sources)	• Use of open–source platforms such as DHIS2 with built in data analysis and visualization aligned with prioritized indicators • Community treatment data integrated into national HIS system to generate treatment ratios (treated over expected cases) by point of service (community/health facility) • Implementing partners and donors support and strengthen the national reporting system • Resources allocated for periodic assessment of ICCM data quality
Information products	• Limited or no procedures in place to regularly transform data into useful information for timely response, priority setting, planning and resource allocation • Limited capacity of staff, especially at district levels, to analyze data	• User–friendly information products (dashboards, reports) analyzing monitoring data for priority indicators produced regularly (at least quarterly) • District level staff with capacity to analyze data and produce information products
Dissemination and use	• Weak linkages to processes for decision–making and corrective actions • Limited tools and training on data use at all levels	• iCCM data use integrated into existing data review and use mechanisms (eg, quarterly review meetings at district level) • Simple tools and training to facilitate data use across levels

M&E – monitoring and evaluation, iCCM – integrated community case management, MOH – Ministry of Health, HIS – health information systems, CHW – community health worker, ICT – information communication technology, DHIS – district health information systems

## WHAT HAVE WE LEARNED ABOUT WHAT IT TAKES TO HAVE A FUNCTIONAL HEALTH INFORMATION SYSTEM FOR iCCM?

As noted, there is no single approach or strategy for how to strengthen routine monitoring for iCCM that will serve all contexts. However, our collective experience in 18 African countries (Cameroon, Cote d’Ivoire, Democratic Republic of Congo (DRC), Ethiopia, Ghana, Guinea, Kenya, Madagascar, Malawi, Mali, Mozambique, Niger, Rwanda, Sierra Leone, South Sudan, Uganda, Zambia, Zimbabwe) involving more than 100 000 CHWs has generated some valuable lessons learned that we believe should inform the necessary efforts to help countries transition towards more functional routine monitoring systems. These lessons and recommendations build on those identified for broader HIS strengthening [4,5]:

**1. Coordination and leadership by Ministry of Health** to develop an overarching framework and rational plans for monitoring and evaluation is necessary to prioritize and harmonize data needs across donors and implementing partners, limit development of parallel systems and promote pooling of resources to strengthen the national system. Interagency technical working groups (TWG) led by the Ministry of Health have proven an effective mechanism in several countries (Malawi, Ethiopia, Sierra Leone, Uganda, Mali, Guinea and Rwanda).With expansion of iCCM, new donors and implementing partners enter the mix and these coordination mechanisms need to be sustained. The ability of TWGs to harmonize monitoring practices is sensitive to the extent to which the MOH exerts leadership and is able to bring partners in line.

**2. Prioritization of a limited number of indicators** that reflect the determinants for achieving high treatment coverage and are tied to specific actions is essential for a routine reporting system to continually generate quality data. The selection and definition of indicators should be informed by global recommendations and the underlying structure and capacity of the health information system [13,14]. **Table 2** outlines data elements that should be captured monthly at district level to generate a minimal set of indicators and identifies other data elements that are better captured periodically through household or CHW surveys. Capturing the number of treatments by CHWs and comparing against the expected number of episodes for each condition based on local epidemiology and care–seeking practices is especially important to understand program performance and identify issues that require further investigation into causes and formulation of appropriate responses [11]. While our experience shows that the data elements required for numerators can be generated even in extremely resource limited settings such as South Sudan, obtaining up to date and accurate information for the denominators (number of children under five in target areas; number of CHWs trained and deployed) remains difficult and requires strategic investments in health workforce tracking.

**Table 2:** Overview of priority data elements for monitoring program performance by frequency of collection

Data elements to capture routinely (monthly)	Data elements best captured periodically (annually or less)
**Core elements required to generate numerators:** • Number of CHWs reporting • Number of CHW treatments by condition • Number of health facility treatments by condition • Number of children referred by CHWs* • Number of CHWs reporting no stock–outs by commodity† • Number of CHW supervision visits conducted **Programs using RDTs should include:** • Number of RDT–tested fevers • Number of RDT+ fevers • Number of treatments for confirmed malaria • Number of treatments for presumptive malaria	**Background data elements required to generate denominators** (update at least annually): • Number of children under–five (overall and in iCCM target areas) • Number of expected cases by iCCM condition (overall and in iCCM target areas) • Number of CHWs trained and deployed to provide iCCM **Data best captured through household or CHW surveys and special studies:** • Gender of cases treated • Follow–up visits for cases treated by CHWs • Referral completion and outcomes • Skills/knowledge of CHWs • Quality of care by provider type (first dose, counseling, use of RDT, use of timer,) • Care–seeking behavior • Timeliness of care–seeking/treatment and source of treatment • Child deaths (total or by cause)

CHW – community health worker, RDT – rapid diagnostic test, iCCM – integrated community case management

*Data on the number of children visiting a CHW during the reporting period must be collected to calculate the referral rate by CHWs

†The iCCM supply chain group recommends collecting three data elements for supply chain management through the Logistics Management Information System (LMIS) for resupply or quantification and monitoring a supply plan: CHW consumption by commodity; stock on hand by commodity; and number of days stocked out during reporting period by commodity.

**3. Early involvement of a representative mix of end users in the development of monitoring tools and systems** contributes to better design and ultimately better quality data. The amount of time and resources required to develop simple, user–friendly tools is often underestimated, leading to sub–optimal or non–functional data collection systems. The CHWs, especially those with lower levels of education/literacy, should be at the center of the development and testing of registers and reporting tools and the same with district managers, facility staff and CHW supervisors for the design of paper–based reports and electronic tools. The design process should aim for **simplicity**, **efficiency** and **scalability** and be suitable for the lowest capacity levels. An example of an overly burdensome approach is the Democratic Republic of Congo, where volunteer, low–literacy CHWs must document details of each consultation using a complex individual sick child form and produce monthly reports containing 98 data elements for iCCM alone. While job aids to guide CHWs through case management protocols are critical, detailed documentation becomes unnecessary and counterproductive once CHWs are acquainted with the algorithm. Apart from setting a double standard by demanding more documentation than required from salaried facility staff, it dilutes data quality and is prohibitively expensive. Integrating use of the tools within initial and refresher trainings and supervision, with sufficient time dedicated for adequate skill–building, is also important.

**4. Integration of community treatment data into national HIS** is critical to allow program managers at various levels to look at treatment data disaggregated by point–of–service to better understand the contribution of iCCM and identify underserved or underperforming areas. The District Health Information Systems (DHIS 2; www.dhis2.org), a free and open source software package, is a promising platform used by a growing number of countries to integrate community information into national HIS. Yet the process is rarely straightforward. The experience from Sierra Leone demonstrates that integration is often lengthy and requires coordination with many stakeholders from different departments and programs [15]. In some countries, such as Mali and Niger, community treatment data are aggregated with health facility treatments making it impossible to distinguish contribution of each point of service. Other countries, such as Malawi, Uganda and Zambia, are unable to calculate treatment ratios because the community and facility sources use different classification systems for childhood illness. Further efforts and targeted investments will be needed to make the necessary progress to fully integrate iCCM data within national HIS.

**5. Strengthening mechanisms for data use and timely response** by program managers, health workers and CHWs requires concerted effort and culture change. Data use requirements are often highest at the district level, where management is in the position to take action. Providing simple tools for data visualization (such as dashboards) and training on data analysis and use promotes improved data quality, enhanced visibility of iCCM services and timely identification and implementation of solutions [16]. To be effective, approaches must focus on a small set of indicators with agreed targets and actionable responses, regularly engage program managers in critical thinking to identify bottlenecks and root causes and address the organization and behavioral determinants of data quality and use [4,5]. An example dashboard developed by PSI for the iCCM program in South Sudan is available on request from the authors and readers are encouraged to consult the paper from IRC on analysis of routine data from six countries for additional guidance [11]. Further work is needed to increase demand for data, integrate data use into existing review mechanisms, and increase accountability of program managers for timely response.

**6. Periodic triangulation of routine data with other data sources** and data quality audits (DQA) should be built into M&E plans to guide interpretation of routine data. Failure to assess quality of routine data early on can lead to false assurances of program performance. Experience shows this is particularly relevant for monitoring CHWs’ skills, quality of care and medicine availability, as data captured through supervision checklists or CHW reports are particularly subject to bias and inter–rater reliability and tends to paint an overly positive picture compared with more structured assessments. For example, analysis of International Rescue Committee (IRC) supervision data from over 170 000 supervision visits in five sub–Saharan countries revealed a pattern of systematic overestimation of the ability of CHWs to correctly count breathing rates (96% of CHWs) when compared with structured assessments (57% of CHWs). These assessments need to be strategically targeted to avoid becoming another data collection burden on an already overstretched system. Participatory, rapid audits of data quality are an effective mechanism to help identify gaps and formulate strategies for improvement and can lead to increased confidence and use of routine data [16]. Reviews of data quality for a subset of the most important indicators can also be built into existing review meetings to institutionalize the process.

**7. Mobile technologies for CHW case management and reporting** can contribute to improved timeliness and availability of data, provided that the basic monitoring system has already been established. The most effective examples are those designed with Ministries of Health and end–users, focused on elements requiring immediate response and linked with platforms such as DHIS2 (for example the mTRAC system in Uganda). There are also good examples of how mobile phone applications have helped connect and motivate CHWs and supervisors by creating closed networks that allow them to communicate at no charge [17]. However, while mHealth solutions offer the potential to streamline reporting and data management procedures, in the short–term they often create an additional burden on CHWs and first level health workers, who are still required to maintain a paper record until mobile applications are widely implemented. Moreover, in many instances, mobile applications have been designed as small scale, resource–intensive projects that proved a distraction rather than a contribution [17,18].

## CONCLUSIONS AND WAY FORWARD

Strong monitoring systems, in which iCCM data are integrated within national health information systems and used to identify issues and take timely action, are essential to improve the ability of iCCM programs to achieve high levels of appropriate utilization and thereby impact child health. In this paper we have characterized the challenges countries face to establish functional monitoring systems for iCCM and outlined some lessons learned based on our experiences in 18 African countries and the literature on strengthening health information systems. As the number of donors funding iCCM and partners supporting implementation continues to grow, it is important to learn from these experiences to avoid repeating common mistakes and to help countries build and sustain functional monitoring systems.

An underlying theme in this paper is that far more attention needs to be paid to the operating environment of iCCM programs when designing monitoring systems. While systems for monitoring iCCM suffer from many of the same shortcomings present with broader health information systems, they do present unique challenges. Community health systems are over–stretched and additional tasks are being shifted from facilities to the lowest level health workers. Implementing iCCM at scale involves thousands of diverse CHWs providing services in the hardest to reach, most deprived communities where formal services have failed to adequately deliver the most basic preventive and curative care. Monitoring systems that expect the most peripheral parts of the health care system to meet rigid reporting timelines, bear the greatest data collection burden, and submit data without consistent, timely and relevant feedback and response will not produce quality information; instead we need to develop systems in which the data collection and reporting requirements are simplified and aligned with the capacity for response, and invest in strengthening mechanisms and accountability for data use.

Going forward, all actors, including the MOH, must shift perspective and consider iCCM as an integral component of the overall health system, including when revising or strengthening monitoring systems and must adapt to the limitations and challenges of the community platform. Donors and implementing partners need to align with national strategic plans for health information systems, including sub–systems such as for iCCM and community health interventions more broadly, harmonize funding toward annual, costed operational plans, streamline and limit routine reporting requirements to the core elements; avoid parallel systems and invest in strengthening routine systems; and provide increased support for institutionalizing capacity of national and district staff for data use and response. Ministries of Health must play a stronger role in coordinating across donors and implementing partners and asserting leadership to better integrate community treatment data into national HIS and to establish mechanisms to increase accountability for data use and response, especially at district and health facility levels.
